# Maternal phylogenetic relationships and genetic variation among Arabian horse populations using whole mitochondrial DNA D-loop sequencing

**DOI:** 10.1186/1471-2156-14-83

**Published:** 2013-09-13

**Authors:** Anas M Khanshour, Ernest Gus Cothran

**Affiliations:** 1 Department of Veterinary Integrative Biosciences, College of Veterinary Medicine and Biomedical Science, Texas A&M University, College Station, TX 77843-4458, USA

**Keywords:** Syrian horse population, Genetic diversity, Arabian horse strains

## Abstract

**Background:**

Maternal inheritance is an essential point in Arabian horse population genetics and strains classification. The mitochondrial DNA (mtDNA) sequencing is a highly informative tool to investigate maternal lineages. We sequenced the whole mtDNA D-loop of 251 Arabian horses to study the genetic diversity and phylogenetic relationships of Arabian populations and to examine the traditional strain classification system that depends on maternal family lines using native Arabian horses from the Middle East.

**Results:**

The variability in the upstream region of the D-loop revealed additional differences among the haplotypes that had identical sequences in the hypervariable region 1 (HVR1). While the American-Arabians showed relatively low diversity, the Syrian population was the most variable and contained a very rare and old haplogroup. The Middle Eastern horses had major genetic contributions to the Western horses and there was no clear pattern of differentiation among all tested populations. Our results also showed that several individuals from different strains shared a single haplotype, and individuals from a single strain were represented in clearly separated haplogroups.

**Conclusions:**

The whole mtDNA D-loop sequence was more powerful for analysis of the maternal genetic diversity in the Arabian horses than using just the HVR1. Native populations from the Middle East, such as Syrians, could be suggested as a hot spot of genetic diversity and may help in understanding the evolution history of the Arabian horse breed. Most importantly, there was no evidence that the Arabian horse breed has clear subdivisions depending on the traditional maternal based strain classification system.

## Background

The fact that the Arabian horse breed is one of the oldest pure breeds and among the most widespread on earth is not disputed [1]. It is the most influential breed throughout the world [2] and has been used to genetically improve several other breeds like the Thoroughbred [3] and the Lipizzan [4]. Furthermore, Western Arabian populations were created in Europe and the USA using stock originally imported from Middle Eastern Arabian populations from sources such as Syria and the Arabian peninsula no longer than 200 years ago [2,5].

The advent of mitochondrial DNA (mtDNA) sequencing in population genetics during the 1970s produced a revolutionary change regarding historical, biogeographic and phylogenetic perspectives on intra- and inter-species genetic structure [6]. Single base pair substitutions have a frequency of up to 10 times higher than nuclear DNA [7]. In addition, strict maternal inheritance of mtDNA [8] and lack of recombination make sequence polymorphism identification a unique application for domestic animals genetic studies not provided by nuclear genes [5]. Therefore, mtDNA has been widely used as a highly informative tool to infer intra- and inter-species phylogenetic relationships [9], and it has successfully been applied to characterize intra-breed variation and the origin of many horse breeds [2,5,10-17]. Furthermore, it can be used to track breed migration and distribution by comparing maternal lines among different populations [18,19].

Unlike the protein-coding gene region, the displacement loop hyper-variable region of mtDNA (D-loop), represented by about 1200 bp, is of particular interest because of its high level of sequence variation [20]. D-loop also is known to be an under-represented mtDNA in Nuclear Mitochondrial sequences (NUMTs) in mammalian species so that it seldom produces NUMTs [21,22]. The D-loop region in horses contains two highly variable segments (HVR1 and HVR2), four conserved blocks (CSB), and variable repeats of 8 bp motif [23,24].

The traditional pattern of breeding Arabian horses affords special opportunities to evaluate variation in matrilineal markers, such as mitochondrial DNA. From a glance of historical records, the Arabian horse breed, in the desert, consists of five strains (*RASANs)* based upon dam line: *Kahlila*, *Saklawia*, *Abiah*, *Shweemat*, and *Muanakii*[1] (some breeders and historians refer to an additional three *RASANs* which are *Hamadania*, *Dahmaa* and *Hadbaa*). The traditional breeders in the Middle East desert (Bedouins) have preserved the purity of the Arabian by avoiding any cross-breeding between the Arabians and non-Arabians and maintaining strictly separated *RASANs*[25]. Consequently, all individuals within a *RASAN* are expected to share the same maternal family line, and they should have similar mtDNA haplotype.

While many studies have been done in horses using mtDNA, only a few included Arabians [2,5,11,26]. Also, the Arabians used were mainly collected from Western populations. Most of the previous studies related to Arabian population genetics used only about 400 bp out of 1200 bp of the mtDNA D-loop. In the present study, we sequenced the whole mtDNA D-loop of Arabian horses collected from the Middle East as well as from Western populations. Our study was designed to investigate the maternal diversity and phylogenetic relationships of Arabian populations and to examine the traditional classification system of the Arabian breed (*RASANs* system) that depends upon maternal family lines.

## Results

Table 1 shows the number of haplotypes (NHap), haplotype diversity (HapD), average number of nucleotide differences (k), the number of polymorphic sites (NPS) and nucleotide diversity (π) for each population. A total of 74 haplotypes from 60 polymorphic sites were found in 271 horses from 11 populations by using the HVR1. NHap increased to 97 using the whole D-loop sequences. Although π decreased from 0.022 to 0.015, NPS increased from 60 to 99 and k increased from 9.7 to 14.5 comparing the HVR1 to the whole D-loop, respectively. The highest HapD values among all tested Arabian populations were in the Syrian, Shagya Arabian and Iranian Arabian populations 0.97, 0.97, 0.96, respectively. The non-Arabian populations also showed high values of HapD, 1.0 in Mongolian and 0.93 in Caspian. All American-Arabian populations -USA-Saudi, USA-Egyptian, USA-Egyptian & Saudi mix and Davenport- showed relatively low HapD ranging between 0.74 and 0.83.

**Table 1:** Populations tested in the study

**Populations (abbreviation)**	**n**	**NHap**		**HapD (SD)**		**NPS**		**π (SD)**		**k**	
		**HVR1**	**W**	**HVR1**	**W**	**HVR1**	**W**	**HVR1**	**W**	**HVR1**	**W**
Syrian (SY)	114	43	50	0.96(0.007)	0.97(0.007)	44	69	0.0196(0.0006)	0.0142(0.0004)	8.6	13.6
Saudi (SU2)	22	10	10	0.84(0.06)	0.84(0.06)	36	50	0.0192(0.0023)	0.0129(0.0015)	8.5	12.4
Iranian Arabian (KA)	10	8	8	0.96(0.06)	0.96 (0.06)	29	42	0.023(0.0019)	0.0153(0.0013)	10.2	14.6
USA-Egyptian(EG)	24	9	9	0.83(0.06)	0.83(0.06)	26	38	0.019(0.0018)	0.0128(0.0012)	8.5	12.1
USA-Egyptian & Saudi mix (SE)	10	4	5	0.79(0.09)	0.84(0.08)	19	26	0.0199(0.0027)	0.0126(0.0015)	8.8	12.1
USA-Saudi (SU1)	31	7	7	0.8(0.042)	0.8(0.042)	34	51	0.0223(0.0016)	0.015(0.001)	9.9	14.3
Shagya Arabian (SA)	9	8	8	0.97(0.06)	0.97(0.06)	30	41	0.0234(0.002)	0.0153(0.0018)	10.3	14.6
Polish Arabian (PA)	13	6	6	0.82(0.08)	0.82(0.08)	25	42	0.0213(0.002)	0.0163(0.0017)	9.4	15.6
Davenport (DV)	19	6	6	0.74(0.083)	0.74(0.083)	26	36	0.020(0.0023)	0.01281(0.0016)	8.9	12.2
Mongolian (MON)	5	5	5	1(0.12)	1(0.12)	19	28	0.0195(0.0038)	0.013(0.0027)	8.6	12.4
Caspian (CS)	14	9	9	0.93(0.045)	0.93(0.045)	35	54	0.023(0.0022)	0.017(0.0013	10.2	16.1
ALL	271	74	97	0.97(0.003)	0.98(0.002)	60	99	0.022(0.0005)	0.0152(0.0003)	9.7	14.5

*n* number of individuals in each population. *NHap* the number of haplotypes resulted in each population. *HapD* Haplotype diversity with its standard deviation. *NPS* the number of polymorphic sites. *π* Nucleotide diversity with its standard deviation. *k* Average number of nucleotide differences. *HVR1* part of the upstream D-loop (450 sites). *W* the whole D-loop (951 sites).

The tested samples were then grouped into strains according to pedigree records and regardless of their populations. We could assign 191 out of 271 samples into seven strains (*RASANs*). Table 2 shows NHap, HapD, k and π for each strain. A total of 44 haplotypes from 52 polymorphic sites were found in these 191 horses of the seven strains using the HVR1 part of the D-loop. The NHap increased to 55 using the whole D-loop sequences. Only *Shweemat* strain had all individuals with a single haplotype. *Hadbaa* and *Dahmaa* also had low NHap, 3 and 2, respectively. *Kahlila* was the most variable strain showing 26 haplotypes. The total NHap calculated from all individuals together (NHap = 55) was less than the sum of NHap calculated from each strain separately due to some shared haplotypes among strains.

**Table 2:** **Strains (*RASANs*) tested in the study**

**Strain (abbreviation)**	**n**	**NHap**		**HapD (SD)**		**NPS**		**π (SD)**		**k**	
		**HVR1**	**W**	**HVR1**	**W**	**HVR1**	**W**	**HVR1**	**W**	**HVR1**	**W**
*Kahlila* (K)	44	22	26	0.94(0.022)	0.95(0.022)	41	61	0.023(0.0006)	0.0149(0.0005)	9.9	14.2
*Hamadania* (H)	61	12	14	0.87(0.019)	0.88(0.02)	32	52	0.019(0.0009)	0.0148(0.0006)	8.4	14.1
*Hadbaa* (HD)	7	3	3	0.76(0.115)	0.76(0.115)	17	24	0.019(0.003)	0.0125(0.002)	8.5	12.0
*Dahmaa* (D)	7	2	2	0.57(0.119)	0.57(0.119)	11	19	0.014 (.0029)	0.0113(0.0023)	6.3	10.8
*Saklawia* (S)	43	14	15	0.92(0.019)	0.92(0.02)	33	48	0.019(0.001)	0.0127(0.0006)	8.4	12.1
*Abiah* (A)	24	10	10	0.85(0.053)	0.85(0.053)	28	39	0.019(0.0015)	0.012(0.001)	8.7	11.7
*Shweemat* (SH)	5	1	1	0	0	0	0	0	0	0	0
all	191	44	55	0.96(0.004)	0.97(0.004)	52	81	0.0218(0.0006)	0.0151(0.0004)	9.9	14.4

*n* number of individuals in each strain. *NHap* the number of haplotypes resulted in each strain. *HD* Haplotype diversity with its standard deviation. *NPS* the number of polymorphic sites. *π* Nucleotide diversity with its standard deviation. *k* Average number of nucleotide differences. *HVR1* part of the upstream D-loop (450 sites). *W* the whole D-loop (951 sites).

**Figure 1:**
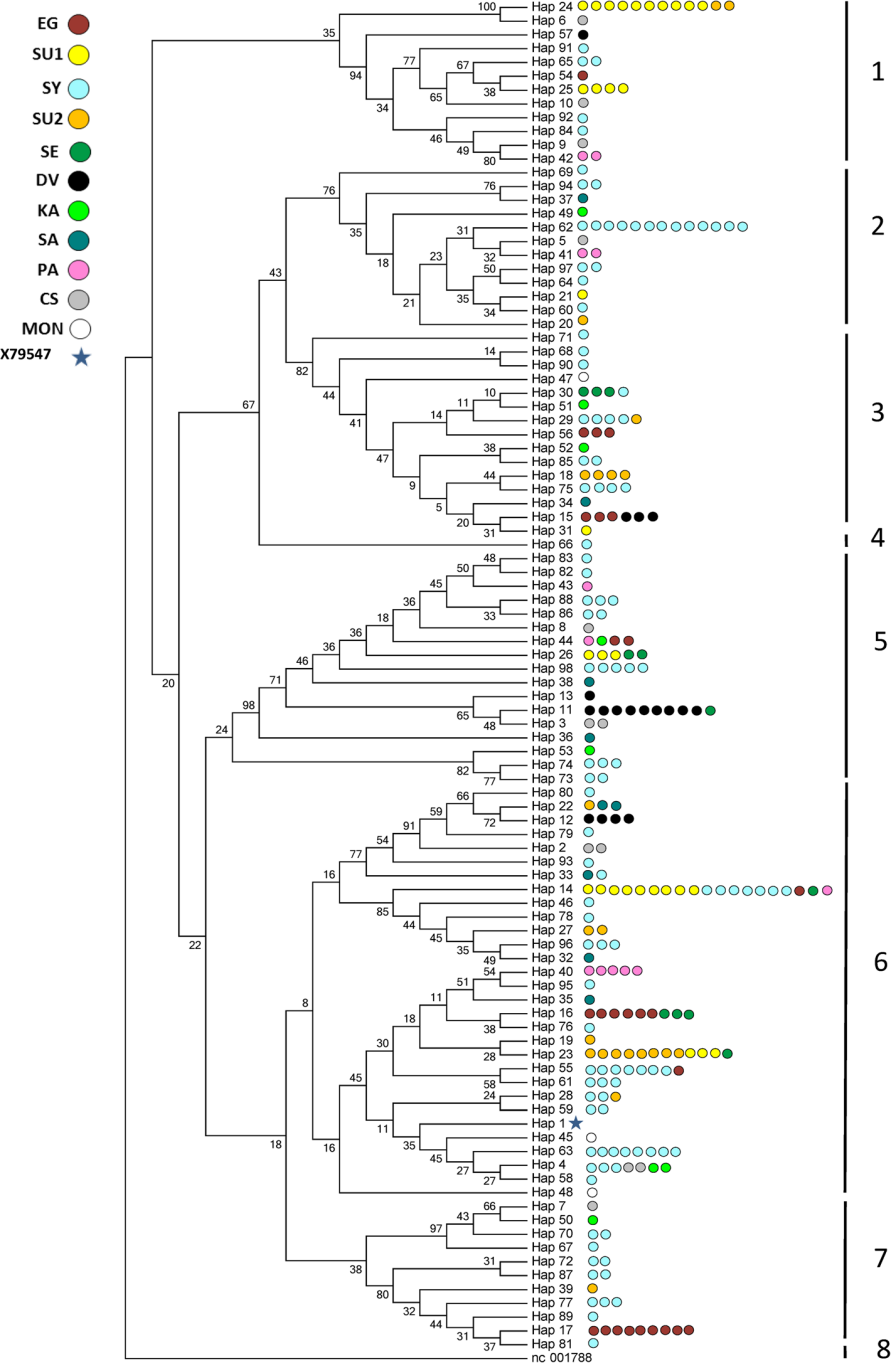
**Consensus Neighbor-joining tree of the 97 haplotypes found.** The tree was drawn based upon 1000 bootstrap replicates. The reference donkey sequence nc 0017788 was used as an out-group. Bootstrap values are shown as percentages. The individuals with each haplotype are represented by colored circles depending on populations. Population abbreviations are found in Table 1.

Figure 1 shows the consensus Neighbor-joining tree of the 97 haplotypes found from all tested populations. No single population was found only in one cluster and different populations shared haplotypes. Fifteen haplotypes: 24, 15, 29, 30, 44, 26, 11, 22, 33, 14, 23, 55, 16, 4 and 28 were found in at least two Arabian populations; for example, haplotype 24 was found in two populations: USA-Saudi and USA-Egyptian; haplotype 14 appeared in five populations: USA-Saudi, Syrian, USA-Egyptian, USA-Egyptian & Saudi mix and Polish Arabian. In addition, haplotype 4 was found in Arabian and non-Arabian populations: Syrian and Iranian Arabian with Caspian. The dendrogram gave seven main clades plus the out-group. Syrian population was the most variable among all populations with individuals found in all clades.

**Figure 2:**
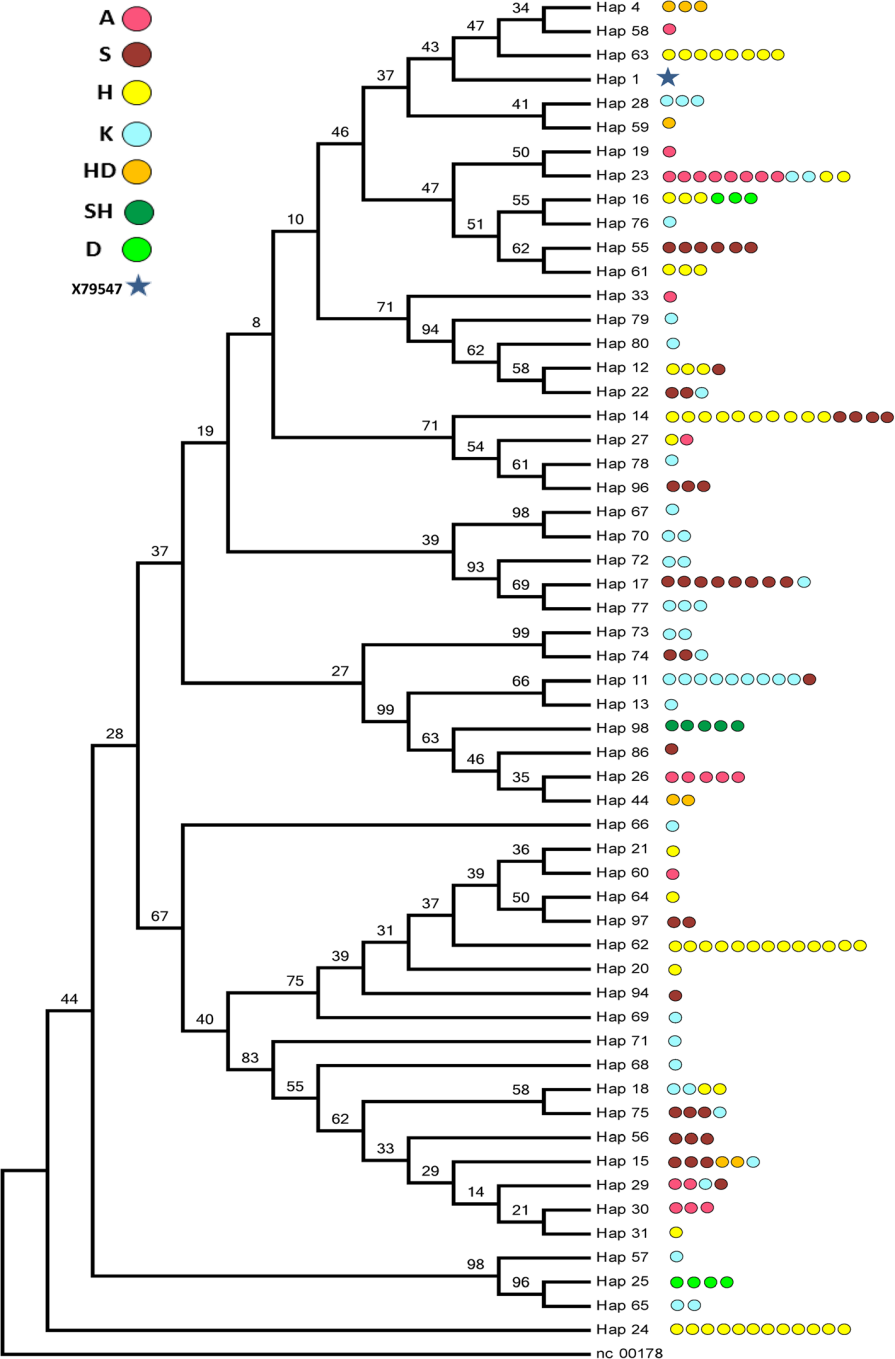
**Consensus Neighbor-joining tree of the 55 haplotypes found in strains.** The tree was drawn based upon 1000 bootstrap replicates. The reference donkey sequence nc 0017788 was used as an out-group. Bootstrap values are shown as percentages. The individuals with each haplotype are represented by colored circles depending on strains. Strain abbreviations are found in Table 2.

Figure 2 shows the consensus Neighbor-joining tree of the 55 haplotypes found in the individuals who were assigned to their strains. None of the tested strains, except *Shweemat*, was represented by a single haplotype or phylogenetically close haplotypes. Each one of the thirteen haplotypes: 16, 23, 22, 12, 27, 14, 29, 15, 18, 75, 11, 74 and 17 was found in at least two strains. For example, haplotype 16 was present in two strains -*Hamadania* and *Dahmaa*- and haplotype 23 in three strains -*Abiah*, *Kahlila* and *Hamadania*-. The most frequent mixing was noticed between *Saklawia* and *Kahlila* strains. The *Kahlila* strain was the most variable among all strains and its individuals were distributed among all clades. The bootstrap values were not high in the trees depicted in Figure 1 and Figure 2. However, a similar pattern of phylogeny resulted by running Bayesian approaches (data not shown).

**Figure 3:**
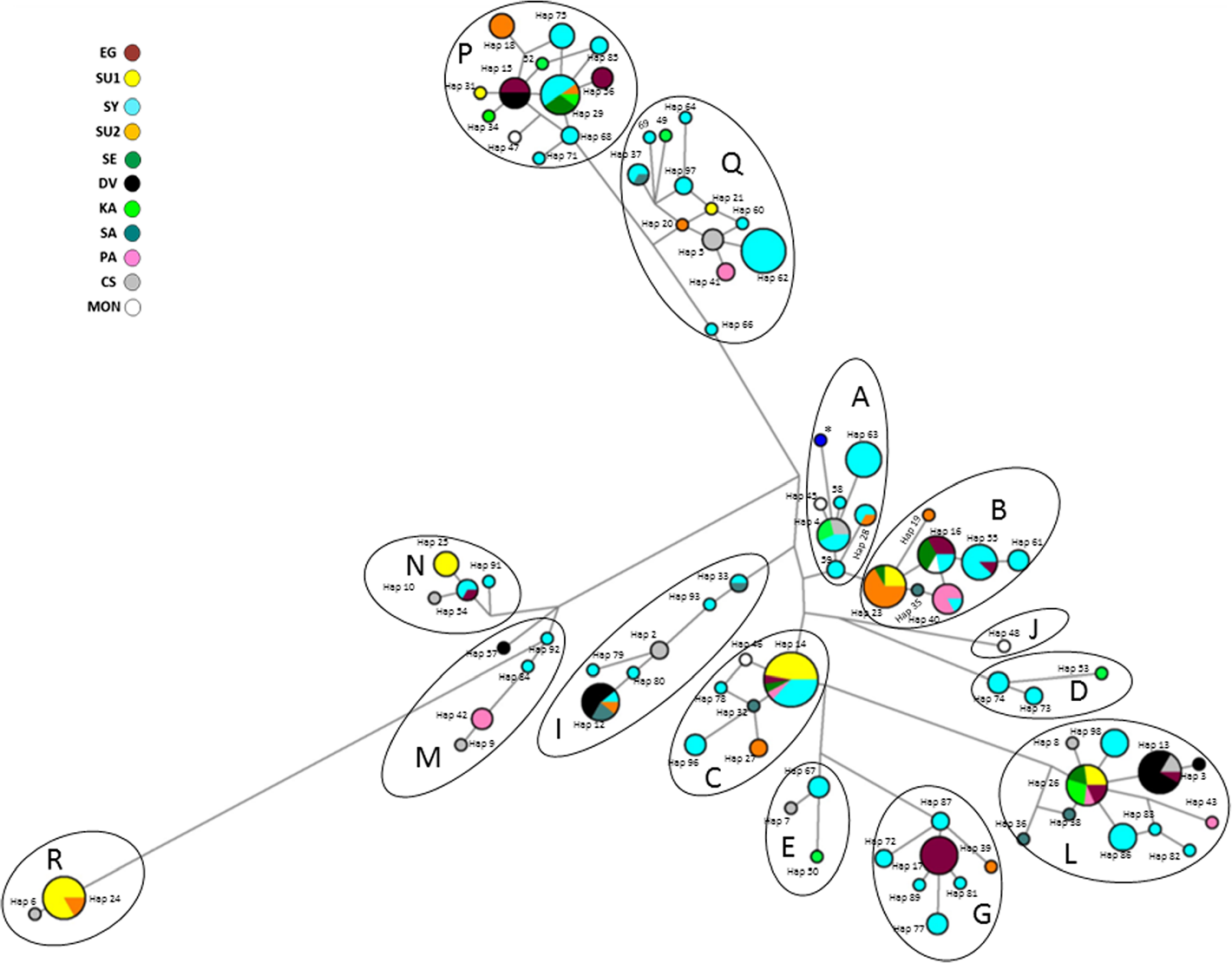
**The median-joining network based on 951 bp of the mitochondrial D-Loop representing 272 horses within 87 haplotypes.** Mutational hot spots were taking into account according to Cieslak et al. [15] and Jansen et al. [16]. The haplogroups were named as defined by Achilli et al. [11]. Each population was shown by color and the proportions of different populations for each haplotype were shown. Population abbreviations are found in Table 1. Reference sample X79547 was labeled with a star.

**Figure 4:**
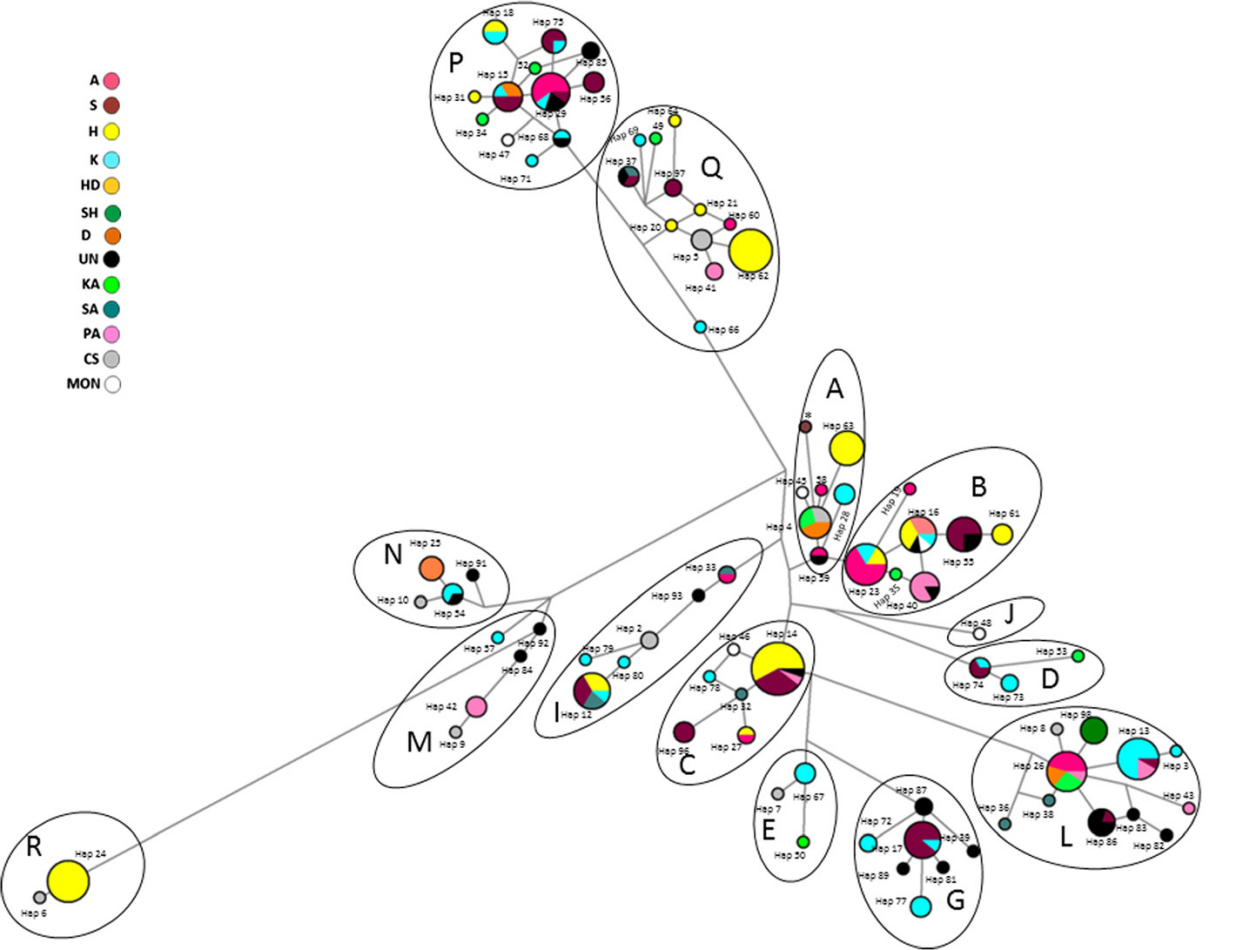
**The median-joining network based on 951 bp of the mitochondrial D-Loop representing 272 horses within 87 haplotypes.** Mutational hot spots were taking into account according to Cieslak et al. [15] and Jansen et al. [16]. The haplogroups were named as defined by Achilli et al. [11] and mentioned as letters next to each haplogroup. Each strain was shown by color and the proportions of different strains for each haplotype were shown. Strain abbreviations are found in Table 2. Samples with unknown strain were represented by black. Reference sample X79547 was labeled with a star.

After taking into account mutational hot spots for the median-joining network (MJ network), the number of haplotypes dropped from 97 to 86. Figures 3 and 4 show the MJ network based on 951 bp of the D-loop representing 271 samples by 86 haplotypes. While in Figure 3 each haplotype is shown by the proportion of the different populations included in this haplotype, in Figure 4 each haplotype is shown by the proportion of different strains. The MJ network showed 14 haplogroups -A, B, C, D, E, G, I, J, L, M, N, P, Q and R- as was defined by Achilli et al. [11].

As shown in Figure 3, each of the 13 haplogroups -A, B, C, D, E, G, I, L, M, N, P, Q and R- contained identical or very close haplotypes from at least two populations. The highest number of populations was found in the haplogroup L. The Arabian populations were represented in all haplogroups except J. The non-Arabian samples were placed in the haplogroups -A, E, I, L, M, N, Q and R- and -A, B, C, J, and P- for the Caspian and the Mongolian populations, respectively. The Syrian population was the most variable with individuals distributed across all haplogroups except J and R. Individuals from the Syrian population had identical or very close haplotypes to individuals from all other Arabian and non-Arabian populations. The Davenport was the least variable Arabian population with only three haplogroups -I, L and P-.

Figure 4 shows that individuals from different strains shared a single haplotype. Identical matching between two or more individuals from different strains was seen in 13 cases. Also, matching was found between known strains and other Arabian groups -Polish Arabian, Shagya Arabian and Iranian Arabian- and non-Arabian populations -Caspian and Mongolian-. In addition, individuals from a single strain were found in distinctive haplogroups (for example: strain *Hamadania* in haplogroups P, C and R). *Kahlila* strain was the most variable with individuals distributed across all haplogroups except J and R. All of the unknown-strain samples were identical or very close to samples of known strains. Although *Shweemat* strain was the least variable with only haplogroup L, it was very close to samples from some other strains.

**Figure 5:**
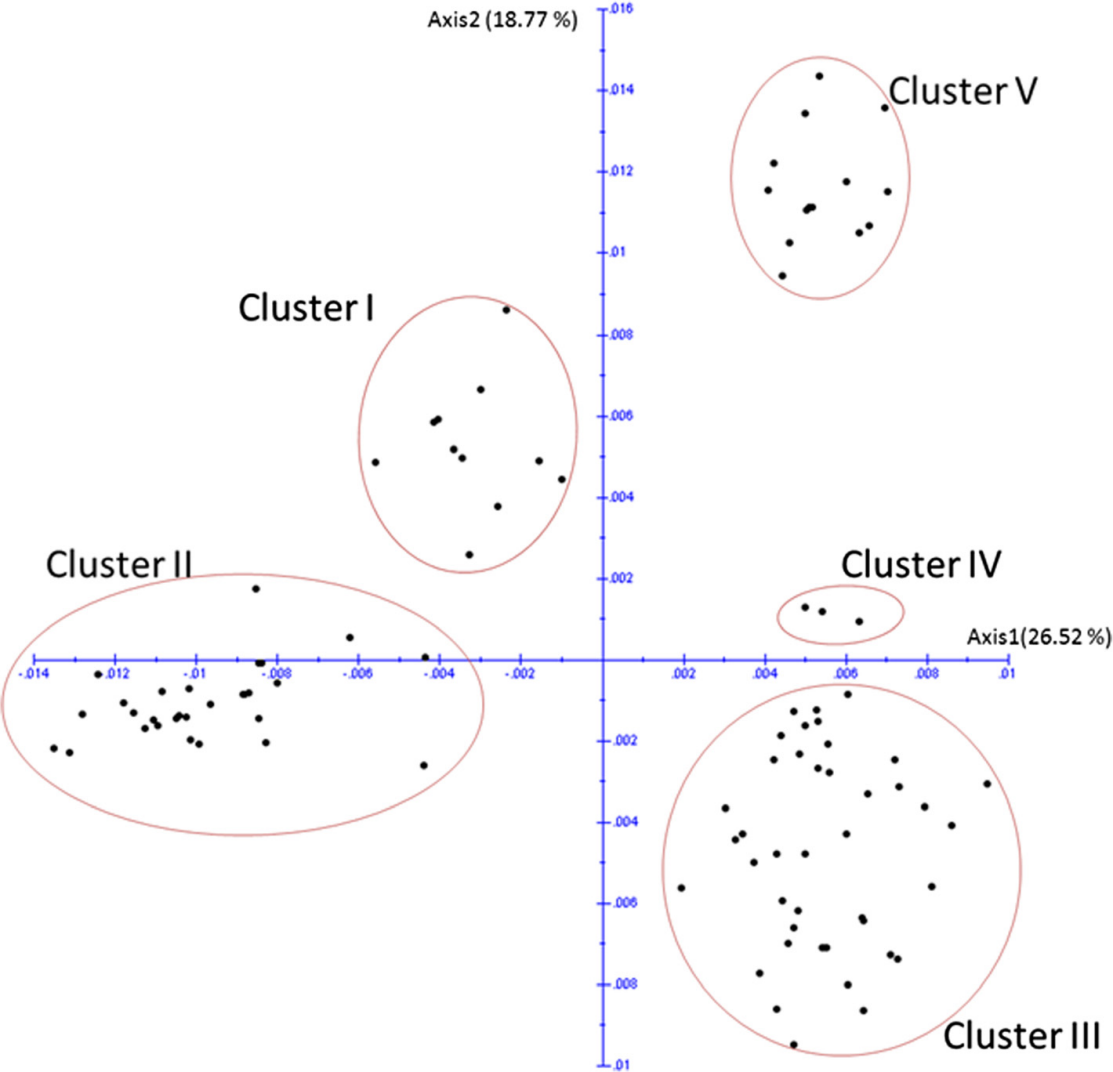
**Plot of the two first axes of the Principal Coordinates Analysis (PCoA) based upon the dissimilarity matrix according to Kimura (1980) based on 951 bp of the 98 haplotype sequences and carried out by using DARwin 5.0 software.** Cluster I includes haplogroups M, N and R. Cluster II includes haplogroups P and Q. Cluster III includes A, B, C, E, G, I, and J. Cluster IV includes haplogroup D. Cluster V includes haplogroup L.

Figure 5 shows the PCoA plot of the two first axes which explain 26.52% and 18.77% of the variability, respectively, and grouped the 98 haplotypes into five clusters. Cluster I included a combination of three haplogroups -M, N and R-. Cluster II consisted of two haplogroups (P and Q). Cluster III included seven haplogroups -A, B, C, E, G, I, and J-. Cluster IV had only haplogroup D, and Cluster V included only haplogroup L. The clustering by PCoA did not show any differentiation among haplotypes that came from different populations (or different strains) but it showed that each cluster contained a mixture of individuals that represented different populations (or strains).

AMOVA showed that the proportion of the variation among populations was 8.25% and the frequency of the variation within populations was 91.75%. The fixation index was equal to 0.083.

The pairwise F_ST_ values are shown in Table 3. Out of 55 pairwise F_ST_ values 28 comparisons had F_ST_ values between 0 and 0.05 showing little genetic differentiation while 21 comparisons had F_ST_ values between 0.05 and 0.15 showing moderate genetic differentiation, six comparisons had F_ST_ values between 0.15 and 0.25 showing great genetic differentiation. Negative F_ST_ values were recorded in some comparisons and these equate to zero F_ST_ values. While most of the lowest F_ST_ values were seen between Syrian and eight other populations -Caspian, USA-Egyptian, USA-Egyptian & Saudi mix, Saudi, Shagya Arabian, Polish Arabian, Mongolian and Iranian Arabian-, the highest F_ST_ values were between the Davenport and five other populations -USA-Egyptian, Saudi, USA-Saudi, Polish Arabian and Mongolian-. None of the comparisons showed values corresponding to very great genetic differentiation.

**Table 3:** **Pairwise F**_**ST **_values among populations*

**Populations**	**CS**	**DV**	**EG**	**SE**	**SU2**	**SU1**	**SA**	**PA**	**MON**	**KA**
DV	0.066									
EG	0.092	0.210								
SE	0.025	0.123	0.024							
SU2	0.076	0.243	0.058	0.033						
SU1	0.057	0.215	0.171	0.125	0.111					
SA	−0.015	0.069	0.066	−0.003	0.051	0.109				
PA	−0.004	0.155	0.092	0.007	0.072	0.104	0.032			
MON	0.027	0.209	0.014	−0.041	−0.040	0.093	−0.017	−0.011		
KA	−0.016	0.045	0.055	−0.029	0.092	0.113	−0.021	0.039	0.029	
SY	0.011	0.149	0.050	−0.001	0.050	0.125	0.015	0.034	−0.009	0.008

*Population abbreviations are found in Table 1.

Negative values equate to zero.

## Discussion

This study presents the first description of maternal genetic diversity based upon the whole mtDNA D-loop of native Arabian horses sampled from Syria, Iran and Saudi Arabia, as well as of Western Arabian populations. One of the unique aspects of this study is the inclusion of the traditional classification system (RASANs or strains system) of native Arabians.

### HVR1 and the whole mtDNA comparison

Most previous maternal diversity studies of horses are based upon sequencing of the HVR1 [5,12,15-17,26-30]. Our results of the comparison between HVR1 and the entire mtDNA D-loop showed that the variability in the upstream region of the D-loop revealed differences among 22 additional haplotypes which had identical sequences in the HVR1. This agrees with Kavar et al. [31] where such a pattern of variability had been found in the D-loop of the Lipizzan horse breed. Higher haplotype diversity (HapD = 0.98) and average number of nucleotide differences (k = 14.5) were found by using the whole mtDNA D-loop compared with the HVR1 (HapD = 0.98 and k = 9.5) (Table 1). Thus, using the whole mtDNA D-loop is more robust and powerful than using the HVR1 alone for analysis of genetic diversity of the mtDNA in horses. Similar results have been reported in goats [32].

### Population genetic diversity

Maternal genetic diversity of the Arabian populations described in this study was similar to that reported in some other breeds [17,33]. Although Syrian, Shagya Arabian and Iranian Arabian populations had equally high HapD values (Table 1), the Syrian population was the most variable based on the consensus Neighbor-joining tree (Figure 1) where the Syrian individuals were found in eight clades compared to the Iranian Arabian and Shagya Arabian individuals found only in five and three clades, respectively. This result also was supported by the MJ-network (Figure 3) where the Syrian population was represented in 12 haplogroups compared to the Iranian Arabian and Shagya Arabian with six and five haplogroups, respectively. According to Achilli et al. [11] there is a total of 18 major haplogroups of horses throughout Asia, Middle East, Europe and America; our results showed that the Syrian population covers 12 of those 18 haplogroups showing a wide maternal genetic diversity. In our opinion, the huge diversity of the Syrian population is not a consequence of recent animal breeding or outcrossing but instead a feature that was already present in this very old population, and this is supported by results of Cieslak et al. [15]. In addition, the huge diversity in Arabian populations is consistent with the multiple origins in the maternal lineages of domestic horse breeds reported by other studies [15,16,28,34]. Some of the Syrian individuals were represented in haplogroup D (Figure 3), haplogroup E according to Jansen et al. [16], that was reported as a very rare and old haplogroup which may date back as far as Bronze age [15,29,35].

The American-Arabian populations showed relatively low HapD values and were represented in a limited number of haplogroups. Davenport population was the least variable with only three haplogroups -I, L and P-. The low maternal diversity found in the American-Arabian populations is probably due to the founder effect. This result is supported by our previous work done by using microsatellite markers where American-Arabian populations showed less genetic variability compared with Middle Eastern populations [36]. Also, Polish Arabians did not show a very high genetic diversity with only 6 haplotypes distributed in four haplogroups. This result did not match with Glazewska et al. [26] where 14 distinct haplotypes were reported. This could be due to sample size or because the horses we used came from close maternal lines.

### Population relationships and genetic structure

The low bootstrap values of the Neighbor-joining trees in Figure 1 and Figure 2 are primarily due to the overall high degree of relationship among horses [37]. Low bootstrap values have been reported in many mtDNA studies in horses [10,14,28,30,38]. Although bootstrap values were low, the populations consistently fell into the same groupings in the trees. The consensus Neighbor-joining tree (Figure 1) and the MJ-network (Figure 3) show that individuals from different populations share identical haplotypes. This indicates possible gene flow among those populations or common ancestry. Identical maternal lines were found between the Syrian and Polish Arabian populations revealing that Syrian mares were probably part of Polish Arabian founders, or some horses were recently introduced to this population. The identical maternal lines that were found between the American Arabian populations -USA-Saudi, USA-Egyptian & Saudi mix and USA-Egyptian- and populations from the Middle East -Syrian, Saudi and Iranian Arabian- confirms that the current registered Arabian horses in America have been primarily founded by mares exported from the Middle East [5]. While Shagya Arabian population is thought to be descended from a Syrian stallion [1], our results show some shared maternal lines between Shagya Arabians and Syrians suggesting a maternal contribution of Syrian horses in Shagya Arabian population, or possibly recent gene flow between these two populations. Furthermore, the phylogenetic analysis revealed that different populations, including Arabian and non-Arabian, often had very close haplotypes, and none of these populations formed a distinct clade. These results together reveal the mixed origin and/or a likely common ancestor of these populations. The genetic clustering analysis using both phylogenic (Figures 1 and 3) and PCoA (Figure 5) did not show any clear pattern of differentiation among all populations. Haplotypes within a population were found in separate haplogroups. Similar results have been reported in other studies of horse mtDNA [10,12,16]. F_ST_ analysis supports this unclear pattern of differentiation showing high rates of mtDNA sharing between populations, with in some cases negative F_ST_ values. F_ST_ values sometimes are produced by software which uses algorithms that include sampling error corrections, such as Arlequin, when the true F_ST_ values are close to zero [39], and usually appear when there are great differences between two random individuals from the same population rather than between two random individuals from different populations [40]. These negative values represent program idiosyncrasies and are effectively zero [41]. AMOVA results also support high within group variation with 91.75% of variability as within population variation.

### Strain relationships and classification system

In the Middle East, strain breeding is still an important factor in the Arabian horse breed [1]. According to Bedouin breeding traditions, Arabian horses were subdivided into strains depending on the maternal lineage. The phylogenetic and principle coordinate analyses in our study using 191 samples, of known strains, showed no evidence that the Arabian breed has clear divisions based upon traditional strain classification. There are four points that support this finding. First, 13 cases revealed that individuals from different strains shared a single haplotype. For example, haplotype 23 was found in individuals that came from three different strains -*Abiah*, *Kahlila* and *Hamadania*-; haplotype 29 was in individuals from three strains -*Abiah*, *Kahlila* and *Saklawia*- (Figure 2). Second, individuals from different strains were found in a single haplogroup. For example, haplogroup P was seen in five strains -*Kahlila, Saklawia, Abiah, Hadbaa,* and *Hamadania*- (Figure 4). Third, each of the strains -*Kahlila, Saklawia, Abiah, Dahmaa, Hadbaa and Hamadania*- was represented in clearly separated haplogroups. For example, *Kahlila* was found in 12 haplogroups (Figure 4). Finally, PCoA did not show any pattern of clustering that fits strains subdivision (Figure 5). Our results agree with the conclusion reported by Bowling et al. [5] about American Arabian horses.

It is possible to have some minor mistakes in the pedigree records of any breed [42], but with our results we can confirm that these mistakes, if they existed in the records that we used, cannot be the reason behind having the huge admixture among tested strains. We do not suspect admixture into the Arabian horse breed, but it is clear that the pedigree records of the Arabian breed were not built using robust genetic tools that can recognize distinct maternal lines in the establishment of the pedigree.

Another important factor in the Bedouin breeding traditions is the sub-strain subdivisions (*MARBATT*) that subdivides each Arabian strain into related groups depending on the tribe’s or owner’s name [1]. Although we did not test the sub-strain subdivisions of Arabians in our study because of a lack of information, we can say that the sub-strain system might be able to partially explain the third point mentioned above (related to the differences among individuals from same strain), but it does not answer the other questions.

## Conclusion

The maternal phylogenetic analysis of native Arabian horses in our study revealed (1) that the whole mtDNA D-loop sequence was more powerful to analyze the genetic diversity in Arabian horses than using just the HVR1, (2) that the maternal genetic diversity was wide in the Arabian horse populations especially in the Syrian population, (3) that there was no clear pattern of differentiation among all tested populations, (4) that the Syrian mares probably had maternal contributions to the Polish Arabian and Shagya Arabian populations, (5) that the current registered Arabian horses in America have been primarily founded by mares exported from the Middle East, and most importantly (6) that there was no evidence that the Arabian breed has clear subdivisions depending on the traditional strain classification system.

## Methods

### Sampling and DNA extraction

Hair samples were collected from 271 horses representing Middle Eastern Arabian, Western Arabian and non-Arabian populations. All tested horses were unrelated from the mothers’ side at least for 3 generations based upon their pedigree records. Tables 1 and 2 show the number of animals used in relation to their populations and strains, respectively. Additional file 1: Table S1 included a summary about populations sampling background and sampling permissions. Total genomic DNA was extracted from hair follicles using the PUREGENE® DNA purification kit following the manufacturer’s instructions.

### Whole D-loop sequencing and data analysis

We designed two pairs of primers based upon the horse mtDNA sequence reference X79547 [23]. We also considered the outcomes reported by Nergadze et al. [43] to minimize the possible amplification of NUMTs that may overlap with D-loop. The designed primers were used to amplify the upstream part between sites 15440 and 16108 (Forward: 5′-AGCTCCACCATCAACACCCAAA-3′. Reverse 5′-CCATG GACTGAATAACACCTTATGGTTG-3′) and the downstream part between sites 16377 and 16642 (Forward 5′-ACCTACCCGCGCAGTAAGCAA-3′. Reverse 5′-AC GGGGGAAGAAGGGTTGACA-3′). Polymerase chain reactions were done for each part separately using the protocol described by Cothran et al. [12]. A total of four sequencing reactions for each sample, including both strands in each part, were carried out using the BigDye® Terminator v1.1 Cycle Sequencing Kit (Applied Biosystems, USA). Sequencing products were purified with BigDye® XTerminator™ Purification Kit (Applied Biosystems, USA). DNA sequences were determined using the ABI 3130 xl Genetic Analyzer (Applied Biosystems, USA). Editing and aligning all sequences were carried out by MEGA 4 [44] using the horse mtDNA sequence X79547 as a reference. Haplotype sequences included in this study were entered into the National Center for Biotechnology Information (NCBI) GenBank database available at http://www.ncbi.nlm.nih.gov/ with the accession numbers [NCBI: KC840701-KC840797]. The statistical quantities for the DNA sequences, including number of haplotypes and haplotype diversity and nucleotide diversity, were carried out using DnaSP 5.10.1 [45]. The statistical analysis was done for each population, as well as for each strain, using two sources of data HVR1, (450 sites) and whole D-loop sequences (951 sites).

Phylogenetic analysis of the haplotypes using a whole D-loop sequence was carried out with the PHYLIP software package [46] based on the Kimura 2-parameter model to calculate genetic distances on the assumption of an equal substitution rate per site [47]. A consensus tree was also constructed with PHYLIP using the Neighbor-joining method [48] with 1000 bootstrap repetitions. The donkey (*Equus asinus*) mtDNA sequence [NCBI: nc_001788] [49] was used as an out-group [10,11].

Another approach for phylog enetic analysis was carried out by drawing the median-joining network (MJ network) [50] in accordance with the haplotype sequences of the whole D-loop using the NETWORK 4.6.1 software (available at http://fluxus-engineering.com). Default settings were applied (r = 2, ϵ = 0) [16], and preliminary trials were done in order to determine the mutational hotspots. Four mutational hot spots were excluded and an additional four were down-weighted into 0.5 [15,16]. Each haplotype in the MJ network was shown by color codes representing the proportions of different strains (or populations) depending on the individual frequencies in each haplotype. Furthermore, the haplotype sequences were compared to the NCBI database using the BLAST search as implemented in MEGA 4, and haplogroups were named as defined by Achilli et al. [11].

To represent the genetic structure and differentiation of tested populations, principal coordinate analysis (PCoA), analysis of molecular variance (AMOVA) and pair-wise F_ST_ were applied. PCoA of the dissimilarity matrix according to Kimura [47] based on 951 bp of the 98 haplotypes sequences was carried out using DARwin 5.0 [51,52]. AMOVA and pair-wise F_ST_ were done using the Kimura 2-parameter model with 1000 permutations and were carried out with Arlequin 3 [53]. For the interpretation of pair-wise F_ST_ results, we followed the suggestion that refers that a value between 0–0.05 indicates little genetic differentiation; a value between 0.05 and 0.15, moderate differentiation; a value between 0.15 and 0.25, great differentiation; and values above 0.25, very great genetic differentiation [54,55].

## Competing interests

We declare that we have no competing interests related to this study.

## Authors’ contributions

AMK and EGC designed the experiment. AMK collected the Syrian Samples, performed the lab works and data analysis and wrote the manuscript. EGC provided the other tested samples, revised the manuscript and supervised the project. AMK and EGC read and approved the final manuscript.

## Supplementary Material

Additional file 1: Table S1 Populations sampling background and sampling permissions. Click here for file
