# Supplementary material for: Maternal phylogenetic relationships and genetic variation among Arabian horse populations using whole mitochondrial DNA D-loop sequencing
Source: BMC Genet. 2013 Sep 13;14:83. doi: 10.1186/1471-2156-14-83 (PMC3847362; doi:10.1186/1471-2156-14-83)
Supplement: Additional file 1: Table S1 — Populations sampling background and sampling permissions. [file 1471-2156-14-83-S1.docx]

| Populations (abbreviation) | n | NHap | | HapD (SD) | | NPS | | π (SD) | | k | |
| --- | --- | --- | --- | --- | --- | --- | --- | --- | --- | --- | --- |
|  |  | HVR1 | W | HVR1 | W | HVR1 | W | HVR1 | W | HVR1 | W |
| Syrian (SY) | 114 | 43 | 50 | 0.96(0.007) | 0.97 (0.007) | 44 | 69 | 0.0196(0.0006) | 0.0142(0.0004) | 8.6 | 13.6 |
| Saudi (SU2) | 22 | 10 | 10 | 0.84(0.06) | 0.84 (0.06) | 36 | 50 | 0.0192(0.0023) | 0.0129(0.0015) | 8.5 | 12.4 |
| Iranian Arabian (KA) | 10 | 8 | 8 | 0.96(0.06) | 0.96 (0.06) | 29 | 42 | 0.023(0.0019) | 0.0153(0.0013) | 10.2 | 14.6 |
| USA-Egyptian(EG) | 24 | 9 | 9 | 0.83(0.06) | 0.83 (0.06) | 26 | 38 | 0.019(0.0018) | 0.0128(0.0012) | 8.5 | 12.1 |
| USA-Egyptian & Saudi mix (SE) | 10 | 4 | 5 | 0.79(0.09) | 0.84 (0.08) | 19 | 26 | 0.0199(0.0027) | 0.0126(0.0015) | 8.8 | 12.1 |
| USA-Saudi (SU1) | 31 | 7 | 7 | 0.8(0.042) | 0.8 (0.042) | 34 | 51 | 0.0223(0.0016) | 0.015(0.001) | 9.9 | 14.3 |
| Shagya Arabian (SA) | 9 | 8 | 8 | 0.97(0.06) | 0.97 (0.06) | 30 | 41 | 0.0234(0.002) | 0.0153(0.0018) | 10.3 | 14.6 |
| Polish Arabian (PA) | 13 | 6 | 6 | 0.82(0.08) | 0.82 (0.08) | 25 | 42 | 0.0213(0.002) | 0.0163(0.0017) | 9.4 | 15.6 |
| Davenport (DV) | 19 | 6 | 6 | 0.74(0.083) | 0.74 (0.083) | 26 | 36 | 0.020(0.0023) | 0.01281(0.0016) | 8.9 | 12.2 |
| Mongolian (MON) | 5 | 5 | 5 | 1(0.12) | 1 (0.12) | 19 | 28 | 0.0195(0.0038) | 0.013(0.0027) | 8.6 | 12.4 |
| Caspian (CS) | 14 | 9 | 9 | 0.93(0.045) | 0.93 (0.045) | 35 | 54 | 0.023(0.0022) | 0.017(0.0013 | 10.2 | 16.1 |
| ALL | 271 | 74 | 97 | 0.97(0.003) | 0.98 (0.002) | 60 | 99 | 0.022(0.0005) | 0.0152(0.0003) | 9.7 | 14.5 |

Table 1: Populations tested in the study. n: number of individuals in each population. NHap: the number of haplotypes resulted in each population. HapD: Haplotype diversity with its standard deviation. NPS: the number of polymorphic sites. π: Nucleotide diversity with its standard deviation. k: Average number of nucleotide differences. HVR1: part of the upstream D-loop (450 sites). W: the whole D-loop (951 sites).
